# Pore- and Core-Scale Insights of Nanoparticle-Stabilized Foam for CO_2_-Enhanced Oil Recovery

**DOI:** 10.3390/nano10101917

**Published:** 2020-09-25

**Authors:** Zachary Paul Alcorn, Tore Føyen, Jarand Gauteplass, Benyamine Benali, Aleksandra Soyke, Martin Fernø

**Affiliations:** 1 Department of Physics and Technology, University of Bergen, 5007 Bergen, Norway; tore.foyen@uib.no (T.F.); Jarand.Gauteplass@uib.no (J.G.); Benyamine.benali@uib.no (B.B.); aleksandra.soyke@uib.no (A.S.); Martin.Ferno@uib.no (M.F.); 2 SINTEF Industry, 7034 Trondheim, Norway

**Keywords:** nanoparticles, foam, CO_2_ EOR, CO_2_ mobility control

## Abstract

Nanoparticles have gained attention for increasing the stability of surfactant-based foams during CO_2_ foam-enhanced oil recovery (EOR) and CO_2_ storage. However, the behavior and displacement mechanisms of hybrid nanoparticle–surfactant foam formulations at reservoir conditions are not well understood. This work presents a pore- to core-scale characterization of hybrid nanoparticle–surfactant foaming solutions for CO_2_ EOR and the associated CO_2_ storage. The primary objective was to identify the dominant foam generation mechanisms and determine the role of nanoparticles for stabilizing CO_2_ foam and reducing CO_2_ mobility. In addition, we shed light on the influence of oil on foam generation and stability. We present pore- and core-scale experimental results, in the absence and presence of oil, comparing the hybrid foaming solution to foam stabilized by only surfactants or nanoparticles. Snap-off was identified as the primary foam generation mechanism in high-pressure micromodels with secondary foam generation by leave behind. During continuous CO_2_ injection, gas channels developed through the foam and the texture coarsened. In the absence of oil, including nanoparticles in the surfactant-laden foaming solutions did not result in a more stable foam or clearly affect the apparent viscosity of the foam. Foaming solutions containing only nanoparticles generated little to no foam, highlighting the dominance of surfactant as the main foam generator. In addition, foam generation and strength were not sensitive to nanoparticle concentration when used together with the selected surfactant. In experiments with oil at miscible conditions, foam was readily generated using all the tested foaming solutions. Core-scale foam-apparent viscosities with oil were nearly three times as high as experiments without oil present due to the development of stable oil/water emulsions and their combined effect with foam for reducing CO_2_ mobility

## 1. Introduction

An energy transition to a net-zero society is a global challenge in need of affordable, low-risk technologies. Carbon capture, utilization and storage (CCUS) is a crucial technology for substantial emission cuts for many energy-intensive industries to achieve the ambitious climate goals of the Paris Agreement [1]. CCUS involves capturing CO_2_ from industrial sources and injecting it into subsurface reservoirs for simultaneous storage and energy production, via CO_2_-enhanced oil recovery (EOR). Permanent CO_2_ storage coupled with CO_2_ EOR can provide affordable and reliable energy for our developing world while reducing the life-cycle carbon emissions of fossil fuels.

CO_2_ EOR has been developed and widely implemented over the past 50 years. CO_2_ is an excellent solvent in EOR processes because it is miscible with most crude oils at reservoir conditions. Above miscibility conditions, CO_2_ swells the oil and reduces its viscosity resulting in increased recovery. Laboratory corefloods have reported high microscopic displacement efficiency and oil recoveries of nearly 100% [2]. However, field-scale operations often report lower than expected recoveries due to poor sweep efficiency and high CO_2_ mobility [3,4]. These issues stem from reservoir heterogeneity and the low viscosity and density of CO_2_ compared to reservoir fluids.

CO_2_ foam can mitigate the impacts of high CO_2_ mobility and reservoir heterogeneity by effectively increasing CO_2_ viscosity, reducing its relative permeability and diverting CO_2_ flow from high permeability zones [5]. CO_2_ foam is generated in porous media by injecting foaming solution with CO_2_, either simultaneously or in alternating slugs. The foam is a dispersion of CO_2_ in liquid where stable liquid films, called lamellae, block some of the pathways for CO_2_ flow [6]. Lamellae are commonly stabilized by surfactants. However, surfactant-stabilized foams can break down in the reservoir due to surfactant adsorption, the presence of oil, and at elevated temperatures and salinities. Therefore, their ability to reduce CO_2_ mobility can be limited. The addition of silica nanoparticles to the surfactant-stabilized CO_2_ foam has been shown to increase the strength and stability of the foam system and provide increased oil recovery [7,8].

Spherical silica nanoparticles are the most commonly used for EOR applications [9]. They are particles with a size up to 100 nm with intrinsic properties different from those found in the bulk of the material due to their high surface-to-volume ratio. Stable emulsions are generated using nanoparticles because a rigid monolayer is formed on the droplet surface and the particles are irreversibly attached to the interface. These emulsions may withstand high-temperature reservoir conditions without agglomeration and the nanoparticles may be further surface-treated to improve stability in harsh conditions. In addition, the small size of the particles, two orders of magnitude smaller than colloidal particles, make them suitable for flow through small pore throats in rock [10,11].

Whether stabilized by surfactants, nanoparticles, or a combination of both, bulk foams are typically composed of bubbles smaller than the containers they are within whereas foam in porous media is composed of bubbles about the same size or larger as the pore space [12]. For foam to generate, lamella creation must exceed lamella destruction. Capillary forces dominate lamella creation by three main mechanisms: leave behind, snap-off and lamella division [5,13].

An issue with foam for EOR applications is the impact of oil on foam (lamellae) stability. Many studies report that oil hinders foam generation and can destabilize already generated foam [14,15,16]. However, these findings are mostly based upon bulk tests at immiscible conditions with surfactant-stabilized foam, which may not necessarily represent foam in porous media and at miscible conditions for CO_2_ and oil. In any case, foam behavior in the presence of oil involves several interactions between the foam, oil, and rock, which may be either detrimental or beneficial to the foam process [17,18]. These interactions include emulsification–imbibition, pseudo emulsions, and entering and spreading [19,20].

In the absence of oil, foam coalescence can reduce the number of bubbles by two mechanisms: texture (bubble size) coarsening by diffusion, often referred to as Ostwald ripening, or capillary suction drainage [21]. Diffusion occurs by the transport of gas from smaller bubbles to larger bubbles, with lower internal pressure, which results in fewer bubbles [22,23]. Capillary suction drainage occurs when the water saturation approaches a saturation value where the lamellae are no longer stable, as the capillary pressure exceeds the maximum disjoining pressure of the foam film and drains the lamellae [24,25].

The majority of earlier work has focused on foam generation and the coalescence of surfactant-stabilized CO_2_ foams in the absence and presence of oil at immiscible conditions. However, much less is known about the role of nanoparticles in the absence and presence of oil at miscible conditions. Thus, this study aimed to thoroughly characterize the dominant foam generation mechanisms and determine the role of nanoparticles for stabilizing CO_2_ foam and reducing CO_2_ mobility. In addition, we shed light on the influence of oil on foam generation and stability. We present a pore- to core-scale characterization of hybrid nanoparticle–surfactant foam formulation for CO_2_ mobility control for CO_2_ EOR and CO_2_ storage. Experimental results compared the hybrid foaming solution to foam stabilized by only surfactant or nanoparticles, in the presence and absence of oil.

## 2. Materials and Procedures

### 2.1. Pore-Scale System

Two foaming agents were used to study foam generation, stability and coalescence. One was a nonionic surfactant (Huntsman *Surfonic L24-22*, Houston, TX, USA), a linear ethoxylated alcohol. The other foaming agent was a surface-modified spherical silica nanoparticle (Nouryon *Levasil CC301*, Amsterdam, The Netherlands). Foaming solutions were made by dissolving each foaming agent, either separately or combined, in 35,000 ppm NaCl brine at the concentrations shown in Table 1. CO_2_ with 99.999% purity was used. The pore space was cleaned between injection cycles using 2-proponal-water azeotrope (IPA). For experiments in the presence of oil, a refined oil (n-Decane, C_10_H_22_) was used to obtain first-contact miscibility with CO_2_.

**Figure 1:**
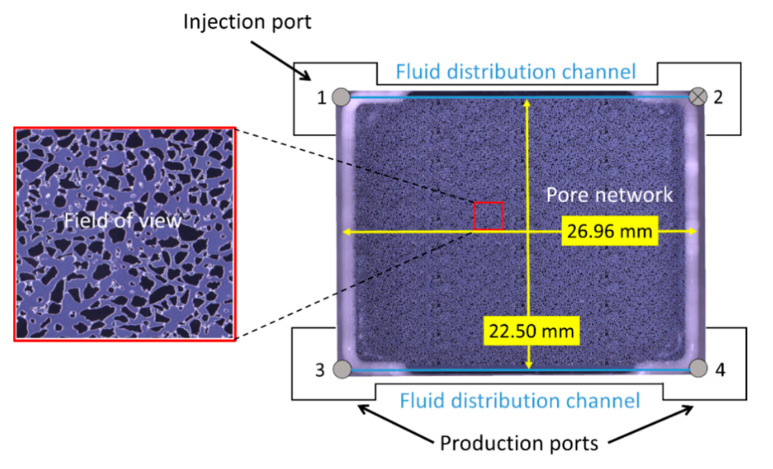
Dimensions of the micromodel, location of the flow ports and the fluid distribution channels. The focused field of view is shown on the left. Injection was into port 1 and production was from ports 3 and 4. Port 2 was closed. The entire pore network consisted of 36 repetitions of a single 749-grain pore pattern. The grain size distribution ranged from 100 to 79,000 ${\mu m}^{2}$ and the pore throat distribution ranged from 10 to 200 μm. The average pore throat length was 89 μm.

The micromodel was composed of a rectangular etched silicon wafer with an irregular porous structure bonded to a transparent borosilicate glass with dimensions of 26.96 mm × 22.50 mm (Figure 1) and a constant etching depth of 30 μm. The pore pattern was a simplified two-dimensional projection of real pore structures with connected pores that allow flow with discontinuous, irregularly shaped grains that provide tortuosity. The chemical composition of the crystalline silicon and borosilicate glass are similar to sandstone and are chemically inert to the injected fluids. Complete manufacturing procedures can be found elsewhere [26,27].

The micromodel had a porosity of 61%, permeability of 3000 mD and pore volume (PV) of 11.1 μL. The porous pattern (27,000 grains) had 36 (4 × 9) repetitions of a pore network with 749 unique grains. The grain size distribution of the 749-grain pattern ranged between 100 and 79,000 ${\mu m}^{2}$ and the pore throat width distribution ranged from 10 to 200 μm. Flow ports were located at each corner of the micromodel with the inlet at ports 1 and 2 and the outlet at ports 3 and 4. The micromodel was positioned in the bottom part of a two-piece polyether ether ketone (PEEK) plastic micromodel holder. The top part had an open window for direct visual observation. The micromodel holder was placed on a motorized stage below a microscope (Axio Zoom. V16, Zeiss, Jena, Germany). The microscope software controlled the zoom, focus, illuminator intensity, imaging, and the motorized stage. Additional details on the micromodel set-up can be found in [28].

### 2.2. Pore-Scale Procedure

The micromodel system was pressurized to 100 bar using a backpressure system at 25 °C for experiments in the absence and presence of oil. For experiments in the absence of oil, foaming solution was first injected to completely saturate the micromodel before injecting dense (liquid) phase CO_2_ at a constant volumetric flow rate of 4 μL/min. The foaming solutions consisted of 1500 ppm nanoparticles, 5000 ppm surfactant, and two hybrid solutions with 5000 ppm surfactant combined with 1500 ppm or 150 ppm nanoparticles. An overview of the foaming solutions are listed in Table 1. A baseline, without foaming solution, was also conducted for comparison. For experiments in the presence of oil, the micromodel was initially saturated with distilled water before injecting six pore volumes of oil. Distilled water was then injected for an additional six pore volumes to achieve residual oil saturation. The micromodel was then saturated with the hybrid 3500 ppm surfactant and 1500 ppm nanoparticle foaming solution before CO_2_ injection began at a constant rate of 1 μL/min. For all experiments, CO_2_ was injected in port 1 (inlet), port 2 was closed and ports 3 and 4 (outlet) were open and kept at 100 bar using the backpressure system (Figure 1). The microscope settings (light intensity, aperture, and shutter time) were optimized for image processing and remained constant. Images were acquired of the entire micromodel with high spatial resolution (4.38 µm/pixel) by stitching multiple overlapping images. The image acquisition time of the porous pattern (121 separate images) was 73 s. A focused field of view was selected, which was representative of the remainder of the micromodel, for detailed analysis and to minimize the capillary end effects. Raw images from the experiments show the grains as dark and opaque and the pore space in a grayish-blue hue. The gas/liquid interfaces (lamellae) were white due to the diffusive ring-illuminator of the microscope. Foam generation and coalescence were also analyzed by utilizing the Python Library OpenCV [29] to identify bubble number and size.

### 2.3. Core-Scale System

The core-scale experiments used the same brine as the pore-scale work. In experiments with only surfactant in the foaming solution, a 3500 ppm or 5000 ppm concentration was used. In experiments with the hybrid foaming solutions, a 3500 ppm surfactant concentration was used with either 1500 ppm or 150 ppm nanoparticles to evaluate the concentration sensitivity for foam stabilization. See Table 1 for an overview of the foaming solutions. A single outcrop Bentheimer sandstone core was used for all experiments to eliminate the impacts of variable core properties. The core was cleaned and dried before being 100% saturated with brine under vacuum. Porosity and pore volumes were calculated based on the weight differential before and after saturation. Absolute permeability was measured between each experiment by injecting brine until a stable differential pressure was obtained for three different injection rates. The permeability of the core was 1400 millidarcy with a porosity of 24% (Table 2).

### 2.4. Core-Scale Procedure

**Figure 2:**
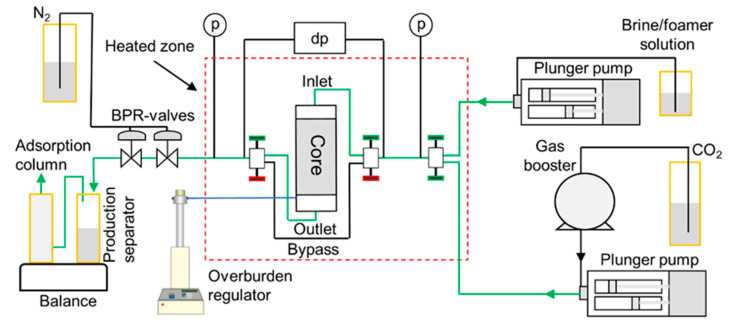
Experimental setup used for the core-scale foam experiments. Green lines indicate the fluid flow directions during the injection of CO_2_ and the foaming solution. Pure CO_2_ was pressurized by a gas booster and injected using a Quizix Q6000-10k plunger pump. Foaming solutions were injected using a Quizix Q5000-10k plunger pump. Injection was performed through a series of needle valves (marked green for open, red for closed) to the top of the core. Produced fluids were depressurized downstream through a series of backpressure regulator (BPR) valves and measured in the production separator and associated water adsorption column using a digital balance. Modified from [30].

The brine-saturated sandstone core was wrapped in a 0.1-mm thick nickel foil to reduce the radial CO_2_ diffusion into the confinement oil before installation into the Viton rubber sleeve. The core was then mounted in a vertically oriented Hassler-type core holder and placed inside a heating cabinet. Experimental conditions were set to 40 °C and 200 bar with a net overburden pressure of 70 bar. At these conditions, CO_2_ is supercritical and has a similar density as in the pore-scale experiments. A differential pressure transducer and two absolute pressure transducers monitored pressure response at the inlet and outlet. Figure 2 shows the experimental set-up, modified from [30].

Foam apparent viscosity is a measure of foam generation, strength and stability. An increase in apparent viscosity indicates a generation of foam and a higher value of apparent viscosity corresponds to a stronger foam. Foam apparent viscosity ($\mu_{app}$) was quantified from the experimental superficial velocities and measured pressure drop [31] by

(1) $$\mu_{app}=\frac{k\nabla p}{\left(u_{l}+u_{g}\right)}$$

where k is the absolute permeability of the porous media, ∇p is the measured pressure gradient and $u_{l}$ and $u_{g}$ are the superficial velocities of liquid and gas, respectively [32]. The effect of nanoparticles on foam strength and stability was evaluated by comparing dynamic experimental apparent viscosity results using foaming solutions with and without nanoparticles.

The injection scheme for the core-scale experiments in the absence of oil was adapted from [33]. First, a minimum of three PVs of foaming solution was injected to satisfy adsorption, displace the initial brine and fully saturate the pore space. Then, CO_2_ was injected from the top of the vertically mounted core at a superficial velocity of 4 ft/day for approximately six PVs. Unsteady state apparent foam viscosities were calculated as a function of time (PVs injected) using Equation (1). A minimum of two experiments were performed for each individual foaming solution. A baseline experiment, without foaming solution, was also conducted for comparison. The core was cleaned between experiments by injecting solutions of IPA before being re-saturated with brine and then foaming solution.

The core-scale procedure in the presence of oil was developed to obtain approximately 30% residual oil before evaluating foam generation and stability. First, a primary drainage with n-Decane for nearly one PV was conducted followed by a waterflood for one PV. Foaming solution was then injected for at least three PVs at a low and high rate. Finally, CO_2_ was continuously injected at 4 ft/day for 10 to 14 PVs. A minimum of two experiments was performed for each individual foaming solution.

## 3. Results and Discussion

### 3.1. Pore-Scale: Foam in the Absence of Oil

**Figure 3:**
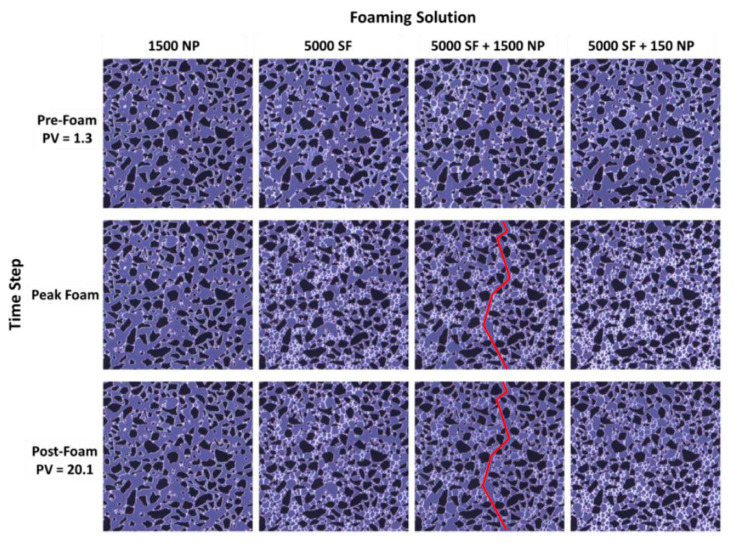
The pore-scale images of a focused field of view during the injection of dense phase CO_2_ into a micromodel saturated with four different foaming solutions at 100 bar and 25 °C. Experiments with different foaming solutions are shown across the top: 1500 ppm nanoparticles (1500 NP), 5000 ppm surfactant (5000 SF), hybrid 5000 ppm surfactant and 1500 ppm nanoparticles (5000 SF + 1500 NP) and hybrid 5000 ppm surfactant and 150 ppm nanoparticles (5000 SF + 150 NP). Injection was from top to bottom in each image. The dark opaque areas are grains, the grayish-blue open areas are the pore space and the thin white films are lamellae. Individual image dimensions are 2190 × 2190 μm. The grain size ranged from 100 to 79,000 ${\mu m}^{2}$ and the pore throat distribution ranged from 10 to 200 μm for the entire micromodel.

Figure 3 shows pore-scale images from four experiments with different foaming solutions. Three time steps are shown which correspond to pre-foam generation (PV = 1.3), peak foam generation and post-foam generation (PV = 20.1). The images show a focused field of view with CO_2_ injection from the top to the bottom for each image. The dark opaque areas are grains, the grayish-blue open areas are the pore space and the thin white films are lamellae.

The experiment with only nanoparticles present (1500 NP) generated weak foam as indicated by the continuous distribution of open flow paths and very few lamellae or bubbles (Figure 3, left column). Thus, CO_2_ mobility remained high and was comparable to the baseline without any foaming agent. CO_2_ injection with the three surfactant-laden foaming solutions resulted in the generation of densely distributed, finely textured foam, which significantly reduced CO_2_ mobility during the peak foam generation stage (5000 SF, 5000 SF + 1500 NP and 5000 SF + 150 NP). Individual bubbles were located near the ends of pore throats and several bubbles filled individual pore bodies, suggesting snap-off as the primary foam generation mechanism. Because the pore bodies had a larger area than the pore throats, repeated snap-off occurred until the pore body was filled with bubbles, a phenomenon also described by [34]. Dynamic observations also revealed many individual lamellae spanning across pore throats. These lamellae may have formed from the leave-behind mechanism because CO_2_ was injected into a surfactant saturated porous media in a drainage-like process. The rise in capillary pressure during drainage can cause lamellae generation by both leave-behind and snap-off as gas enters the pore network [35].

Direct visual observations of the experiment with the hybrid foaming solution containing 5000 ppm surfactant and 1500 ppm nanoparticles revealed a continuous open flow path for CO_2_ throughout the duration of the experiment (Figure 3, red line, 5000 SF + 1500 NP). No lamellae impeded CO_2_ flow in this region and the CO_2_ relative permeability was reduced by the presence of lamellae in the remainder of the pore network. Therefore, within this focused field of view, a continuous gas-foam was generated.

**Figure 4:**
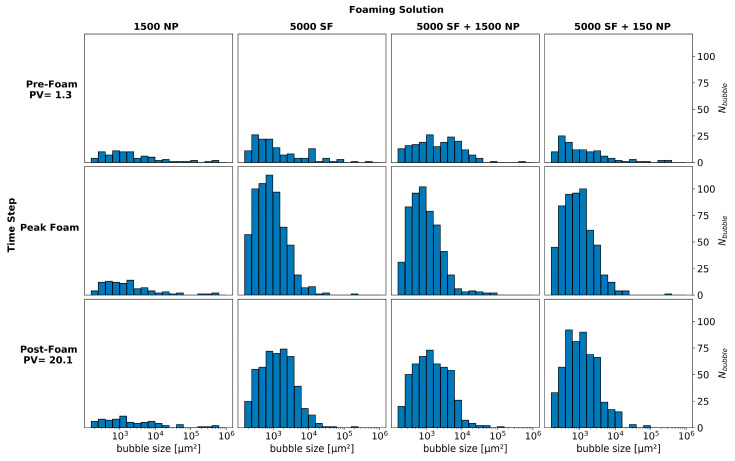
The number of bubbles (N_bubble_) versus bubble size for the micromodel experiments with four different foaming solutions. Foaming solutions are shown across the top and include 1500 ppm nanoparticles (1500 NP), 5000 ppm surfactant (5000 SF), hybrid 5000 ppm surfactant and 1500 ppm nanoparticles (5000 SF + 1500 NP) and hybrid 5000 ppm surfactant and 150 ppm nanoparticles (5000 SF + 150 NP).

Figure 4 quantifies the number of bubbles versus the bubble size for the images shown in Figure 3. Bubble number and size were used as indications of foam generation and strength where a higher bubble number corresponded to a finer textured foam. All foaming solutions containing surfactant-generated small bubbles (≤10^3^ µm^2^) at the peak generation stage. In the post-foam generation stage, the total number of bubbles decreased and their size increased; hence, the foam texture coarsened, increasing CO_2_ mobility as CO_2_ was continuously injected. The hybrid foaming solutions with either 1500 ppm or 150 ppm nanoparticles showed similar behavior, indicating that foam strength and stability was not sensitive to nanoparticle concentration when used together with the selected surfactant.

**Figure 5:**
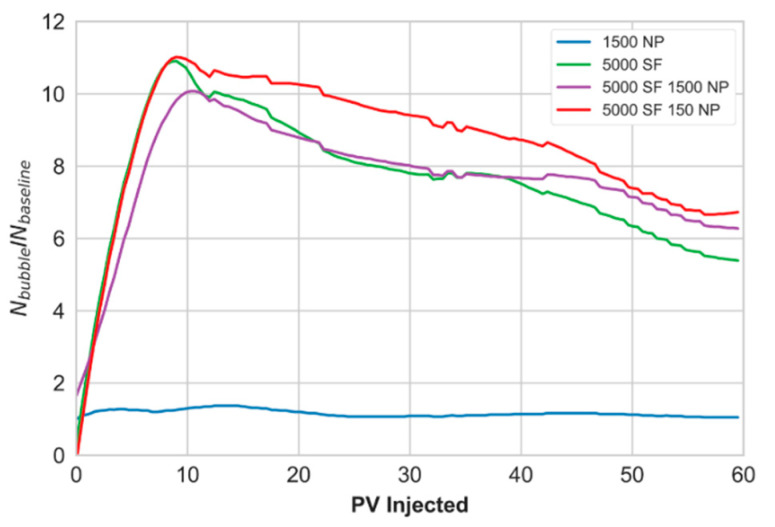
Development in normalized bubble number as a function of pore volume (PV) injected using four different foaming solutions for the focused field of view. The blue curve represents the foaming solution with 1500 ppm nanoparticles (1500 NP), the green curve represents the 5000 ppm surfactant solution (5000 SF), the purple curve represents the hybrid solution with 5000 ppm surfactant and 1500 ppm nanoparticles (5000 SF + 1500 NP) and the red curve represents the hybrid solution with 5000 ppm surfactant and 150 ppm nanoparticles (5000 SF + 150 NP).

Pore-scale foam behavior was also analyzed by examining the total bubble number (N_i_) as a function of the PV of CO_2_ injected. The number of bubbles during foam generation and coalescence (N_bubble_) were normalized to baseline (N_baseline_) for the four foaming solutions. Figure 5 shows the normalized bubble number as a function of PV injected for each foaming solution for the focused field of view. Foam generation (as indicated by bubble number) increased from approximately 9 to 11 times the baseline for all foaming solutions. Peak foam generation was reached after approximately seven PVs of the CO_2_ injected. After peak foam generation, the number of bubbles steadily decreased from bubble coarsening as the dominant coalescence mechanism as observed in Figure 3. The hybrid foaming solutions, containing nanoparticles and surfactant, had a limited impact on the number of bubbles and foam stability during continuous CO_2_ injection.

The two-dimensional geometry of the micromodel likely resulted in multiple bubbles per pore because the widths of some of the pore throats were narrower than the pore throat depths. Therefore, pore-scale foam texture may not have a direct relation to foam in three-dimensional porous media. Many studies report that in situ foam usually consists of bubbles about the same size or larger than pore bodies based upon effluent analysis during laboratory experiments and the large flow resistance for bubbles smaller than pores [12,36,37]. In addition, most mechanistic foam models [38,39,40] assume a single bubble per pore and that discrete bubbles flow through the porous media, where foam strength is controlled by foam texture (bubble size). The latter assumptions are supported by the pore-scale observations reported here.

### 3.2. Pore-Scale: Foam in the Presence of Oil

**Figure 6:**
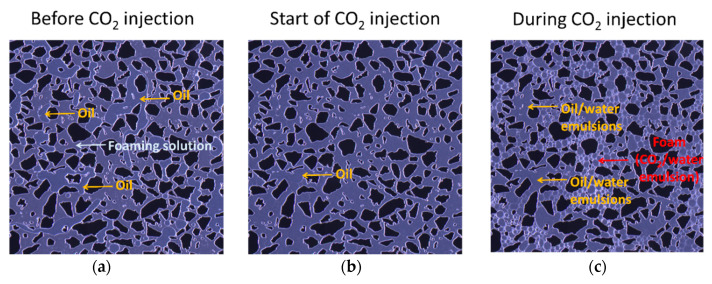
Pore-scale images of a focused field of view during the injection of dense phase CO_2_ into a micromodel saturated with a hybrid foaming solution and oil at 100 bar and 25 °C. Three stages of the experiment are shown which correspond to: (**a**) before CO_2_ injection; (**b**) the start of CO_2_ injection; and (**c**) during CO_2_ injection. Injection was from top to bottom in each image. The dark opaque areas are grains, the grayish-blue open areas are the pore space filled and the thin white films are the lamellae. Individual image dimensions are 2190 × 2190 μm. The grain size ranged from 100 to 79,000 ${\mu m}^{2}$ and the pore throat distribution ranged from 10 to 200 μm for the entire micromodel.

Dynamic foam generation in the presence of oil was evaluated by injecting CO_2_ into a micromodel saturated with a hybrid foaming solution and oil. The aim was to evaluate the impact of oil on foam generation and gain insight on the influence of oil/water emulsions during CO_2_ foam processes. Figure 6 shows the pore-scale images of the unsteady-state CO_2_ injection in the presence of oil with the hybrid foaming solution containing 3500 ppm surfactant and 1500 ppm nanoparticles. Three stages of the experiment are shown which correspond to before CO_2_ injection, the start of CO_2_ injection, and during CO_2_ injection. Each image was acquired with 75 s between each time step.

Before CO_2_ injection, the micromodel was initially saturated with foaming solution and oil (Figure 6a). Foaming solution appears as the continuous liquid phase, whereas oil is seen as isolated globules in interconnected pores. At the start of CO_2_ injection (Figure 6b), the oil globules faded due to miscibility between CO_2_ and oil. As CO_2_ injection continued, the oil was displaced by CO_2_ and foam readily generated in areas where oil was not present. Oil not displaced formed oil/water emulsions and occupied pores without foam present (Figure 6c). The foam (CO_2_/water emulsion) had thicker lamellae compared to the oil/water emulsions likely due to interfacial tension differences at these conditions as also observed in [41]. Compared to foam (CO_2_/water emulsion) alone, the combined effect of oil/water emulsions and foam further reduced CO_2_ mobility. This resulted in increased “foam” strength as also observed in the core-scale experiments in the presence of oil (discussed in Section 3.4).

### 3.3. Core-Scale: Foam in the Absence of Oil

**Figure 7:**
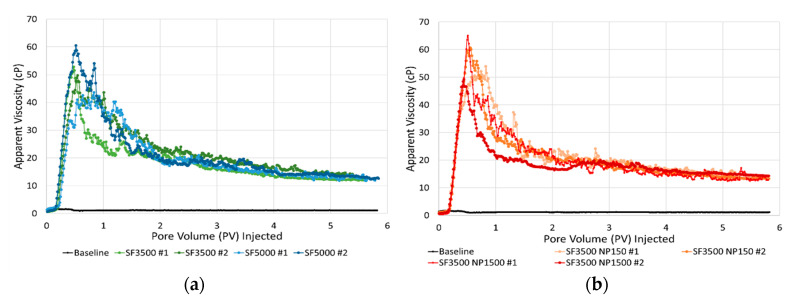
Apparent viscosity versus pore volume of the CO_2_ injected for the unsteady state CO_2_ injections into cores pre-saturated with foaming solutions containing: (**a**) 3500 ppm surfactant (green curves) and 5000 ppm surfactant (blue curves); (**b**) hybrid foaming solutions containing 3500 ppm surfactant and 150 ppm nanoparticles (orange curves) and 3500 ppm surfactant and 1500 ppm nanoparticles (red curves). The black curve is the baseline with only brine.

Dynamic foam generation and stability in the absence of oil was evaluated by injecting CO_2_ into cores saturated with different foaming solutions. This set of experiments established conditions to investigate foam behavior during prolonged periods of CO_2_ injection in a drainage-like process. Figure 7a shows the apparent viscosity versus pore volume of CO_2_ injected for the CO_2_ foam stability scans with foaming solutions containing only surfactant at concentrations of 3500 ppm (green curves) and 5000 ppm (blue curves). Figure 7b shows the results from the experiments using the two hybrid foaming solutions with 3500 ppm surfactant and 150 ppm nanoparticles (orange curves) and 3500 ppm surfactant and 1500 ppm nanoparticles (red curves). A baseline scan, without foaming solution, is also shown in each figure for comparison (black curves).

For all experiments, the rapid and linearly increasing apparent viscosity until 0.2 PV injected indicated that foam was generated as CO_2_ invaded the core saturated with foaming solution. Apparent viscosity steadily increased, from 0.2 to 0.5 PV injected, as foam continued to generate and propagate into the core. A peak in apparent viscosity (foam strength) was achieved after approximately 0.5 PV was injected. The magnitude of the peak apparent viscosity varied from 45 to 65 cP for all experiments. The peak in apparent viscosity indicated a transition from a period of predominantly foam generation to predominantly foam coalescence. The development of a continuous CO_2_ flow path not impeded by lamellae caused the foam to coalesce, likely related to a combination of bubble rupture and foam displacement. The CO_2_ flow path rapidly reduced the apparent viscosity just before one PV was injected. After about six PVs were injected, the initial CO_2_ viscosity was not fully recovered due to trapped bubbles in the pore space, which continued to reduce CO_2_ mobility.

The difference in dynamic foam generation and coalescence processes for the foaming solutions with and without nanoparticles were insignificant. Including nanoparticles in the surfactant-laden foaming solution did not result in a more stable foam and the type of foaming solutions did not clearly affect the apparent viscosity of the foam. Therefore, the surfactant contributed mostly to foam generation and the nanoparticles had only minor impacts on the foam strength and stability in these experiments. The experiments with the hybrid foaming solutions (Figure 7b) revealed similar foam behavior independent of nanoparticle concentration. Despite an order of magnitude difference in nanoparticle concentration, the measured apparent viscosities and stability of the foam were similar. Thus, the nanoparticle concentrations of 150 ppm gave similar performance as the nanoparticle concentrations of 1500 ppm when used with the selected surfactant. The next set of experiments focused on evaluating the same foaming solutions in the presence of oil, a condition known to destabilize some surfactant-based foams.

### 3.4. Core-Scale: Foam in the Presence of Oil

**Figure 8:**
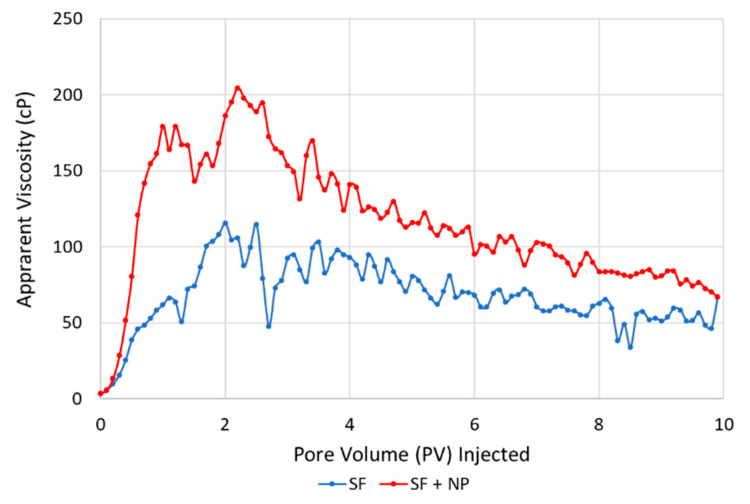
Apparent viscosity versus pore volume of the CO_2_ injected for the unsteady state CO_2_ injections in cores with residual oil (S_or_) and pre-saturated with a hybrid foaming solution containing surfactant and nanoparticles (SF + NP, red curve) or a foaming solution containing only surfactant (SF, blue curve).

Dynamic foam generation and stability for foaming solutions with and without nanoparticles in the presence of oil was evaluated by injecting CO_2_ into a core saturated with each foaming solution. The core contained a residual oil saturation of around 30% prior to being flooded with foaming solution and then CO_2_. Each experiment was conducted a minimum of two times for reproducibility. Figure 8 shows the average apparent viscosity (cP) versus the pore volume of CO_2_ injected for the unsteady state CO_2_ foam stability scans in the presence of oil. Experiments using the foaming solution with only surfactant are shown with the blue curve and experiments with the hybrid foaming solution are shown with the red curve.

Both types of foaming solutions generated foam within the first 0.2 PV injected. However, the hybrid foaming solution generated foam more rapidly (faster increase in apparent viscosity) than the solution containing only surfactant. In addition, the hybrid foaming formulation generated a stronger (higher apparent viscosity) foam, compared to the solution containing only surfactant. The increased apparent viscosity for both types of solution indicated that each formulation generated foam with the residual oil present.

Apparent foam viscosity values with the hybrid solution in the presence of oil (Figure 8, red curve) were nearly three times as high as the experiments without oil present (Figure 7b). In the presence of oil, the foaming solution with only surfactant (Figure 8, blue curve) had foam-apparent viscosity values about twice as high as experiments in the absence of oil (Figure 7a). This is related to the development of oil/water emulsions, which were likely stabilized by each respective foaming agent. The emulsions influenced the calculated apparent viscosities (differential pressure) and are indistinguishable from foam (CO_2_/water emulsion). Nonetheless, the oil/water emulsions highlight an important facet of the CO_2_ foam process, which can be beneficial to enhancing oil recovery by increasing the capillary number (increased viscous forces and lower interfacial tension) [42].

### 3.5. From Pore- to Core-Scale

The similarity in foam generation and coalescence during unsteady-state CO_2_ injections at the pore- and core-scale is striking. Figure 5 and Figure 7 reveal dynamic foam generation and coalescence processes with similar behavior at two different length scales. The experiments in this work were characterized by a period of rapid foam generation during drainage-like CO_2_ injection and a period of foam coalescence during prolonged CO_2_ injection. The decline in foam strength, at both scales, was related to the development of open CO_2_ flow paths through the generated foam. This phenomenon was a result of bubble coarsening from diffusion. The pore-scale observations unlocked real-time insights on in situ foam behavior that may help explain the observations from the core-scale experiments. Since foam was rapidly generated at both scales (due to ideal conditions for foam generation), the coalescence mechanisms during continued CO_2_ injection at the pore-scale may be applied at the core-scale with some level of confidence. It is understood that foam will dry out as more CO_2_ is injected and not supplemented with additional surfactant solution. Here, we showed one of the physical mechanisms responsible for such behavior.

In addition, the experiments in the presence of oil revealed the importance of stable oil/water emulsions on the CO_2_ foam process. The insights from pore-scale experiments with oil shed light on the influence of oil/water and CO_2_/water emulsions on CO_2_ mobility reduction. Higher foam apparent viscosities were calculated for the core-scale experiments with oil present and were likely related to the development of the oil/water emulsions. Because apparent viscosity is used as an indication of foam generation and strength in laboratory experiments, care must be taken when interpreting the results from coreflood studies with the presence of stable oil/water emulsions. These emulsions can influence the calculated apparent viscosities (based on differential pressures) and may contribute to reducing CO_2_ mobility.

## 4. Conclusions

This work presented a multi-scale investigation of hybrid nanoparticle–surfactant foam for CO_2_ mobility control for CO_2_ EOR and CO_2_ storage. High-pressure micromodel experiments and high-pressure/high-temperature core floods evaluated a hybrid surfactant and nanoparticle foaming solution and foaming solutions with only surfactant or nanoparticles, in the presence and absence of oil. The following conclusions can be drawn:

- Direct pore-scale observations of dense phase CO_2_ injection into a micromodel saturated with foaming solutions containing only surfactant or a hybrid nanoparticle–surfactant foaming solution revealed snap-off as the primary foam generation mechanism and leave-behind as a secondary foam generation mechanism.
- At the pore-scale, foam readily generated in areas where oil was not present and oil/water emulsions initially occupied pores without foam present.
- All foaming solutions containing surfactant generated foam in the presence and absence of oil, whereas foaming solution only containing nanoparticles did not. Thus, surfactant was the main foam generator and nanoparticles may be more important for foam stabilization.
- Foam strength was not sensitive to nanoparticle concentration when used together with surfactant in the tested foaming solutions.
- At the core-scale, all foaming solutions rapidly generated foam in the presence of residual oil.
- Foam apparent viscosity values with the hybrid foaming solution, in the presence of oil, were nearly three times as high as the experiments without oil. This was related to the development of oil/water emulsions, which were likely stabilized by the foaming agents.
- A link is proposed between direct pore-scale visual observations and quantitative core-scale measurements. The combined influence of stable oil/water emulsions and foam (CO_2_/water emulsions) may be beneficial for increasing the capillary number by achieving higher apparent viscosity and lower interfacial tension.
- The experiments in this work were characterized by a period of rapid foam generation during drainage-like CO_2_ injection and a period of foam coalescence during prolonged CO_2_ injection. The decline in foam strength is related to the development of open CO_2_ flow paths through the generated foam.
- Increased apparent viscosities with foam reduced CO_2_ mobility at multiple length scales, which can improve volumetric sweep efficiency in field-scale CO_2_ EOR and CO_2_ storage processes.

## Figures and Tables

**Table 1:** Composition of the foaming solutions used in pore- and core-scale experiments.

Foaming Agents	Concentration, *Component*	Scale
Nanoparticle (NP)	1500 ppm, *Levasil CC301*	Pore
Surfactant (SF)	3500 ppm, *Surfonic L24-22*	Core
	5000 ppm, *Surfonic L24-22*	Pore and Core
Hybrid (SF + NP)	3500 ppm, *Surfonic L24-22* + 1500 ppm, *Levasil CC301*	
	5000 ppm, *Surfonic L24-22* + 1500 ppm, *Levasil CC301*	Pore
	5000 ppm, *Surfonic L24-22* + 150 ppm, *Levasil CC301*	
	3500 ppm, *Surfonic L24-22* + 150 ppm, *Levasil CC301*	Core

**Table 2:** Core properties of the Bentheimer sandstone used in the experimental work.

Core Properties	Value
Length (cm)	24.6 ± 0.01
Diameter (cm)	3.64 ± 0.01
Pore Volume (mL)	68.23
Porosity	0.24
Permeability (mD)	1400
